# Association Between Recommended Preconception Health Behaviors and Screenings and Improvements in Cardiometabolic Outcomes of Pregnancy

**DOI:** 10.5888/pcd18.200481

**Published:** 2021-01-21

**Authors:** Kaitlyn K. Stanhope, Michael R. Kramer

**Affiliations:** 1 Department of Gynecology and Obstetrics, Emory University School of Medicine, Atlanta, Georgia; 2 Department of Epidemiology, Emory University, Atlanta, Georgia

## Abstract

**Introduction:**

Gestational diabetes (GDM) and hypertensive disorders of pregnancy (HDP) are associated with increased risk of maternal and infant illness and long-term elevated cardiometabolic risk. Little information exists on the prevention of either disorder before pregnancy. Our goal was to describe the association between preconception indicators and risk of gestational diabetes and hypertensive disorders of pregnancy.

**Methods:**

We used logistic regression to analyze cross-sectional data from the 2016–2017 Pregnancy Risk Assessment Monitoring System (N = 68,493) to quantify the association between 14 preconception health indicators (across domains of health care, nutrition and physical activity, tobacco and alcohol, chronic conditions, mental health, and emotional and social support) and, separately, GDM and HDP. We accounted for sampling weights and controlled for maternal age, race/ethnicity, prepregnancy insurance, prepregnancy body mass index, and report of a check-up in the year before pregnancy.

**Results:**

Prepregnancy obesity was the strongest predictor of both HDP (adjusted odds ratio [aOR], 3.1; 95% CI, 2.8–3.5) and GDM (aOR, 3.1; 95% CI, 2.7–3.5). Individual behaviors (eg, exercise, attending a check-up) were not associated with either HDP or GDM. A diagnosis of diabetes before pregnancy predicted HDP (aOR, 2.3; 95% CI, 1.7–3.0).

**Conclusion:**

Prepregnancy chronic disease and obesity predicted pregnancy complications (ie, GDM and HDP). Given the challenges in reversing these conditions in the year before pregnancy, efforts to improve preconception health may be best directed broadly to expand access to primary care for all women.

**Summary**

**What is known on this topic?**

Risk of gestational diabetes and hypertensive disorders of pregnancy vary by maternal race/ethnicity, family history, and body mass index.

**What is added by this report?**

Women’s behaviors in the year before pregnancy were not predictive of gestational diabetes or hypertensive disorders of pregnancy in the 2016–2017 Pregnancy Risk Assessment Monitoring System. However, chronic disease status (obesity, diabetes, or hypertensive diagnosis) was associated with increased risk.

**What are the implications for public health practice?**

Efforts to improve preconception health may be best directed broadly to expand access to primary care for all women.

## Introduction

Since the early 1990s, pediatricians, obstetricians, and public health practitioners have focused on promoting preconception health to improve pregnancy outcomes (1,2). Preconception health encompasses physical, mental, and social health indicators and allows for assessing population-level well-being among individuals of reproductive age (3). For individuals, preconception health is often operationalized as recommended behavior changes in the year before pregnancy (4). For example, the Centers for Disease Control and Prevention (CDC) recommends that women attend preventive health visits, achieve a healthy weight, maintain control of chronic diseases, meet recommended levels of exercise, and avoid drinking or smoking (2). Evidence about how women’s individual behaviors in the year before pregnancy contribute to maternal outcomes is limited (5).

Rates of hypertensive disorders of pregnancy (HDP) and gestational diabetes (GDM) have increased over the past 2 decades, particularly among women of racial and ethnic minorities. In this population, rates of HDP increased from 3.8% in 2001 to 8.0% in 2019, and rates of GDM increased from 3.7% in 2000 to 6.9% in 2019 (6–8). GDM and HDP are associated with increased risk of maternal illness and death (9,10) and increased risk of cardiovascular disease (11–13). Evidence for the effectiveness of preconception behavior changes for preventing GDM or HDP is sparse (14,15). The only behavior with strong evidence of effectiveness is control of preexisting diabetes to reduce risk of HDP, which applies only to the small number of women with chronic diabetes (16,17). Although preconception obesity is associated with increased risk of both GDM and HDP (18,19), no evidence indicates that weight loss interventions in the year before pregnancy (eg, diet, exercise) reduce risk of either HDP or GDM (14,15,20).

Identifying risk factors for GDM and HDP modifiable in the year before pregnancy would provide individuals planning their pregnancies with a way to prevent adverse maternal and cardiovascular outcomes. The goal of this article is to describe the association between preconception health indicators and risk of GDM and HDP.

## Methods

### Study population

We used data from all sites participating in phase 8 of the Pregnancy Risk Assessment Monitoring System (PRAMS), comprising a cross-sectional, representative sample of births for each included site (state or US territory) for years 2016–2017. CDC releases sites with at least 55% response rates; 39 sites during 2016–2017 had a sufficient response rate. Each site participating in PRAMS randomly selects women who have had a live birth in the past year to complete a survey about their preconception, prenatal, and postpartum behaviors, health outcomes, and experiences (21). PRAMS is weighted to give a representative sample of all women who gave birth in participating sites in those years. For this analysis, we included all women who had complete information on gestational diabetes, gestational hypertension, or both, as well as all included covariates In total, 8.1% of observations were excluded because of missing data (n = 1,105 excluded for missing information on GDM or HDP; n = 4,945 excluded for missing race/ethnicity or insurance). The final analytic sample of unweighted observations was 68,493. Compared with women who were included in the analysis, women who were excluded were more likely to have public insurance before their pregnancy (39.1% [SE, 0.26] vs 29.1% [SE, 1.84]) and less likely to report non-Hispanic white race/ethnicity (42.7% [SE, 1.32] vs 56.9% [SE, 0.27]). For analyses of GDM, we excluded an additional 2,617 participants who reported pregestational diabetes (analytic data set: n = 65,876 unweighted observations). For analyses of associations with HDP, we exclude an additional 4,350 participants reporting pregestational hypertension (analytic data set: n = 64,143 unweighted observations).

### Measures

Our outcomes were 1) whether a woman had experienced GDM during this pregnancy and 2) whether a woman had experienced HDP during this pregnancy. Previous research has shown low positive predictive value (~50%) for self-report of pregnancy complications on PRAMS (22). Thus, for GDM and HDP, we used data from the birth certificate, which is linked to PRAMS survey responses for all participants. Both GDM and HDP have acceptable (>80%) positive predictive values on the birth certificate when compared with medical record diagnoses (23).

CDC considers 38 preconception health indicators, 23 of which are measured on PRAMS (3). Of these, we excluded 8 indicators that applied only to multiparous women or reflected postpartum recommendations and 2 for which we did not have data (ie, emotional abuse and use of assisted reproductive technology) resulting in 13 indicators across 7 domains: health care (health-care coverage in the month before pregnancy; and routine check-up, teeth cleaning, and advice to improve health before pregnancy in 12 months before pregnancy); pregnancy intention; tobacco and alcohol use (any smoking or drinking in 3 months before pregnancy); nutrition and physical activity, including prepregnancy body mass index (BMI: underweight [<18.5 kg/m^2^], normal weight [18.5–24.9 kg/m^2^], overweight [25.0–29.9 kg/m^2^], or obese [≥30.0 kg/m^2^]), folic acid supplementation, and exercising at least 3 times per week in 3 months before pregnancy); mental health (clinical care for depression or anxiety in 12 months before pregnancy); emotional and social support (physical abuse in 12 months before pregnancy); and chronic conditions (diabetes or hypertension diagnosis at any time before pregnancy).

### Analysis

All analyses were conducted using SAS survey procedures version 9.4 (SAS Institute) or SUDAAN (RTI International) to account for survey weights. To understand sources of variation in the prevalence of GDM and HDP, we first calculated the prevalence of GDM and HDP across maternal demographic and socioeconomic characteristics and the prevalence of each preconception recommendation across maternal race/ethnicity. Because of the small number of Hawaiian/Pacific Islander women who met inclusion criteria, we do not present estimates on them, although they are included in the total number of women in the final sample. For multivariable analyses, we grouped American Indian/Alaska Native women, Hawaiian/Pacific Island women, Asian women, and Other/Mixed women.

To estimate associations between recommended preconception indicators and GDM/HDP, we fit logistic models between each indicator and each outcome (GDM/HDP) separately. We present adjusted odds ratios (ORs) and 95% CIs. For GDM, we excluded women who reported a diagnosis of diabetes before pregnancy. For HDP, we excluded women who reported a diagnosis of hypertension before pregnancy. Because maternal preconception health indicators and perinatal risk are correlated with women’s socioeconomic status, including demographics and access to care, we selected certain variables a priori for adjustment. In each model, we adjusted for maternal age (≤17, 18–19, 20–24, 25–29, 30–34, 35–39, ≥40), race/ethnicity (non-Hispanic white, non-Hispanic black, Hispanic, non-Hispanic other), prepregnancy insurance (private, public, or none), prepregnancy BMI, and whether the woman reported attending a check-up with an obstetrician/gynecologist (OB/GYN) or primary care physician before pregnancy.

## Results

Overall, 8.4 of women reported an HDP and 6.2% reported GDM (Table 1). The prevalence of both conditions varied most by race/ethnicity, age, and educational level, and both conditions were more common among older women. HDP were most common among non-Hispanic Black women (12.6%), whereas GDM was most common among American Indian/Alaska Native women (13.1%). No clear pattern was found for HDP by maternal education. However, women with the least education (<8th grade) were the most likely to report a diagnosis of GDM (8.5% compared with 6.4% among women with a college degree or higher).

**Table 1:** Prevalence of Hypertensive Disorders of Pregnancy and Gestational Diabetes, by Socioeconomic Status (N = 68,493), Pregnancy Risk Assessment Monitoring System, United States, 2016–2017

Variable	Hypertensive Disorders of Pregnancy	Gestational Diabetes
	Prevalence, % (SE)	
**Total**	8.4 (0.16)	6.2 (0.14)
**Maternal education**		
<8th grade	6.3 (0.77)	8.5 (0.98)
Grades 9–12, no diploma	8.0 (0.50)	4.7 (0.38)
High school graduate	8.8 (0.36)	5.8 (0.31)
Some college	9.7 (0.32)	6.7 (0.28)
College degree and beyond	7.4 (0.24)	6.4 (0.23)
**Marital status**		
Married	7.9 (0.19)	6.9 (0.19)
Unmarried	9.2 (0.28)	5.0 (0.22)
**Maternal age, y**		
≤17	7.0 (1.40)	0.3 (0.16)
18–19	8.9 (0.97)	1.6 (0.31)
20–24	7.4 (0.37)	3.0 (0.24)
25–29	7.5 (0.27)	5.3 (0.24)
30–34	8.7 (0.30)	7.5 (0.29)
35–39	9.7 (0.41)	10.0 (0.45)
≥40	13.6 (1.04)	12.4 (1.12)
**Maternal race/ethnicity**		
Non-Hispanic White	8.2 (0.20)	5.5 (0.18)
Non-Hispanic Black	12.6 (0.52)	5.5 (0.37)
Asian	11.4 (0.95)	8.7 (0.76)
American Indian/Alaska Native	5.0 (0.43)	13.1 (0.81)
Other/mixed	8.4 (0.80)	5.7 (0.70)
Hispanic/Latina	6.9 (0.36)	7.3 (0.84)
**Birth year**		
2016	8.2 (0.23)	5.2 (0.19)
2017	8.7 (0.22)	5.9 (0.20)

Preconception indicators across all domains varied by maternal race/ethnicity (Table 2). Most women did not receive advice about improving their health before pregnancy (70.7%). Asian and non-Hispanic White women were the most likely to have insurance 1 month before conception and non-Hispanic White women were more likely to attend a check-up or have a teeth cleaning 1 year before conception. Hispanic women were the least likely to have insurance (35.4% uninsured). Non-Hispanic Black women (38.7%) were the most likely to report the pregnancy as unintended, followed by American Indian/Alaska Native women (27.2%). Non-Hispanic White women were more likely to report drinking prior to conception and engaging in protective behaviors (vitamin use or exercise) compared with any other racial/ethnic group. American Indian/Alaska Native women were the most likely to report a diagnosis of depression in the year before pregnancy (18.7% compared with 12.6% overall). Non-Hispanic Black women were more likely to report a prepregnancy diagnosis of hypertension compared with all other groups (8.6% compared with 5.2% overall).

**Table 2:** Prevalence of Self-Reported Preconception Health Indicators, by Race/Ethnicity (N = 68,493), Pregnancy Risk Assessment Monitoring System, United States, 2016–2017

Health Indicator	Total	AI/AN	Asian	Hispanic	NHB	NHW	Other/Mixed
	% (SE)						
**Health Care**							
Received advice about improving health (1 y before conception)	29.3 (0.32)	31.9 (1.95)	33.7 (1.40)	32.9 (0.92)	34.6 (0.97)	27.1 (0.39)	28.3 (1.72)
Check-up (OB/primary care) (1 y before conception)	62.9 (0.35)	59.0 (2.23)	56.7 (1.47)	55.0 (1.01)	51.1 (1.04)	67.9 (0.42)	56.1 (2.00)
Teeth cleaning (1 y before conception)	62.9 (0.35)	59.0 (2.23)	56.7 (1.47)	55.0 (1.01)	51.1 (1.04)	67.9 (0.42)	56.1 (2.00)
Health insurance (1 mo before conception)							
Private	56.4 (0.29)	16.7 (1.61)	72.7 (1.04)	31.7 (0.68)	37.4 (0.80)	69.0 (0.37)	46.6 (1.58)
Medicaid/other public	29.1 (0.26)	76.4 (1.64)	18.5 (0.83)	32.9 (0.65)	50.1 (0.81)	22.3 (0.33)	42.4 (1.55)
None	14.5 (0.22)	6.9 (0.72)	8.8 (0.78)	35.4 (0.72)	12.6 (0.58)	8.7 (0.24)	11.0 (1.02)
**Reproductive Health**							
**Pregnancy intention**							
Intended	59.4 (0.29)	47.0 (1.56)	68.6 (1.09)	54.9 (0.74)	38.3 (0.80)	65.6 (0.38)	52.6 (1.59)
Ambivalent	15.1 (0.22)	25.8 (1.37)	12.5 (0.73)	13.8 (0.50)	23.0 (0.68)	13.7 (0.28)	17.7 (1.18)
Unintended	25.5 (0.27)	27.2 (1.34)	18.9 (0.94)	31.3 (0.69)	38.7 (0.80)	20.7 (0.33)	29.6 (1.45)
**Nutrition and physical activity**							
Folic acid (1 mo before conception)	48.4 (0.30)	33.8 (1.40)	55.7 (1.17)	38.0 (0.72)	36.6 (0.79)	54.4 (0.39)	45.6 (1.59)
Exercise 3x weekly (1 y before conception)	42.9 (0.58)	34.9 (3.45)	37.0 (2.54)	45.2 (1.54)	32.3 (1.51)	45.9 (0.73)	38.5 (3.21)
Body mass index (kg/m^2^)	8.0 (0.17)	5.2 (0.57)	12.9 (0.78)	15.6 (0.53)	7.7 (0.45)	5.1 (0.17)	7.0 (0.86)
Underweight (<18.5)	44.6 (0.30)	33.5 (1.34)	59.3 (1.15)	35.0 (0.70)	33.8 (0.77)	49.3 (0.39)	41.5 (1.53)
Normal (18.5–24.9)	24.4 (0.26)	26.0 (1.36)	19.4 (0.91)	26.6 (0.67)	25.4 (0.70)	23.7 (0.33)	27.5 (1.47)
Overweight (25.0–29.9)	23.1 (0.25)	35.4 (1.61)	8.4 (0.67)	22.9 (0.61)	33.1 (0.77)	21.9 (0.32)	24.0 (1.35)
Obese (≥30.0)	23.1 (0.25)	35.4 (1.61)	8.4 (0.67)	22.9 (0.61)	33.1 (0.77)	21.9 (0.32)	24.0 (1.35)
**Tobacco and alcohol use**							
Drinking (3 mo before conception)	57.3 (0.30)	48.9 (1.58)	33.3 (1.10)	40.8 (0.73)	46.4 (0.82)	68.2 (0.36)	57.0 (1.57)
Smoking (3 mo before conception)	17.4 (0.23)	37.6 (1.56)	4.3 (0.41)	9.5 (0.42)	16.9 (0.61)	21.1 (0.33)	23.2 (1.40)
**Mental health status**							
Depression/anxiety diagnosis (1 y before conception)	12.6 (0.20)	18.7 (1.46)	4.9 (0.57)	8.6 (0.40)	11.2 (0.50)	14.9 (0.28)	14.8 (1.10)
**Chronic conditions**							
Diabetes (3 mo before conception)	3.5 (0.11)	4.5 (0.60)	5.1 (0.58)	3.8 (0.28)	3.5 (0.27)	3.2 (0.14)	3.1 (0.52)
Hypertension (3 mo before conception)	5.2 (0.13)	8.3 (0.87)	4.3 (0.52)	4.2 (0.29)	8.6 (0.43)	4.8 (0.17)	5.5 (0.74)
**Emotional and social support**							
Physical abuse (1 y before conception)	1.7 (0.08)	4.3 (0.76)	0.8 (0.20)	1.7 (0.18)	2.8 (0.29)	1.4 (0.09)	2.7 (0.48)

Abbreviations: AI/AN, American Indian/Alaska Native; NHB, non-Hispanic Black; NHW, non-Hispanic White; OB, obstetrician.

Adjusting for all covariates (age, race/ethnicity, insurance, BMI, prepregnancy check-up), the strongest preconception predictor of GDM was prepregnancy obesity (aOR, 3.1; 95% CI, 2.7–3.5) followed by receiving prepregnancy advice to improve health compared with not receiving advice (aOR, 1.4; 95% CI, 1.2–1.5), and lack of insurance (aOR, 1.2; 95% CI, 1.0–1.4) (Table 3). In adjusted models, other individual behaviors (exercising before pregnancy, drinking before pregnancy, a prepregnancy check-up, or taking folic acid) were not associated with decreased risk of GDM.

**Table 3:** Associations Between Self-Reported Preconception Health Indicators and Gestational Diabetes or Hypertensive Disorders of Pregnancy, Pregnancy Risk Assessment Monitoring System, United States, 2016–2017^a^

Health Indicator	Gestational Diabetes (N = 65,876)		Hypertensive Disorders of Pregnancy (N = 67,950)	
	Crude OR (95% CI)	Adjusted OR (95% CI)	Crude OR (95% CI)	Adjusted OR (95% CI)
**Health Care**				
Received advice about improving health (1 y before conception)	1.5 (1.3–1.7)	1.4 (1.2–1.5)	1.2 (1.1–1.4)	1.1 (1.0–1.3)
Check-up (OB/primary care) (1 y before conception)	1.1 (1.0–1.2)	1.0 (0.9–1.1)	1.2 (1.1–1.3)	1.2 (1.1–1.3)
Teeth cleaning (1 y before conception)	0.9 (0.8–1.0)	1.0 (0.8–1.1)	0.8 (0.7–0.9)	0.8 (0.7–0.9)
Health insurance (1 mo before conception)				
Private	[1 Reference]			
Medicaid or other public	0.9 (0.8–1.0)	1.1 (0.9–1.2)	1.0 (0.9–1.1)	0.9 (0.8–1.0)
None	1.1 (0.9–1.2)	1.2 (1.0–1.4)	0.8 (0.7–0.9)	0.9 (0.7–1.0)
**Reproductive Health**				
**Pregnancy intention**				
Intended	1 [Reference]			
Ambivalent	0.8 (0.7–0.9)	0.8 (0.7–1.0)	1.0 (0.9–1.2)	1.0 (0.9–1.1)
Unintended	0.7 (0.6–0.8)	0.8 (0.7–1.0)	1.0 (0.9–1.1)	0.9 (0.8–1.0)
**Nutrition and physical activity**				
Folic acid (1 mo before conception)	1.0 (0.9–1.1)	0.9 (0.8–1.0)	1.0 (0.9–1.1)	1.0 (0.9–1.1)
Exercise 3x weekly (1 y before conception)	1.1 (0.9–1.4)	1.1 (0.9–1.4)	0.9 (0.8–1.1)	0.9 (0.8–1.1)
Prepregnancy BMI (kg/m^2^)				
Underweight (<18.5)	1.4 (1.1–1.7)	1.3 (1.1–1.6)	1.0 (0.8–1.3)	1.1 (0.9–1.4)
Normal (18.5–24.9)	1 [Reference]			
Overweight (25.0–29.9)	1.7 (1.5–2.0)	1.7 (1.5–2.0)	1.8 (1.6–2.1)	1.8 (1.6–2.1)
Obese (≥30.0)	3.0 (2.6–3.3)	3.1 (2.7–3.5)	3.1 (2.8–3.5)	3.1 (2.8–3.5)
**Tobacco and alcohol use**				
Drinking (3 mo before conception)	0.9 (0.8–1.0)	0.9 (0.8–1.0)	1.1 (1.0–1.2)	1.0 (0.9–1.1)
Smoking (3 mo before conception)	1.0 (0.8–1.1)	1.1 (0.9–1.2)	1.1 (1.0–1.2)	1.1 (0.9–1.2)
**Mental health status**				
Depression/anxiety diagnosis (1 y before conception)	1.0 (0.9–1.2)	1.1 (0.9–1.3)	1.2 (1.0–1.4)	1.1 (0.9–1.2)
**Chronic conditions**				
Hypertension diagnosis (3 mo before conception)	1.6 (1.3–2.0)	1.1 (0.9–1.4)	—	—
Diabetes diagnosis (3 mo before conception)	—	—	2.8 (2.1–3.7)	2.3 (1.7–3.0)
**Emotional and social support**				
Physical abuse (1 year before conception)	0.9 (0.6–1.3)	0.9 (0.6–1.4)	1.2 (0.8–1.8)	1.2 (0.8–1.7)

Abbreviations: —, not applicable; BMI, body mass index; OB, obstetrician; OR, odds ratio.

a Adjusted for check-up in the year before pregnancy, race/ethnicity, age, BMI, and preconception insurance.

Similarly, prepregnancy obesity was the strongest preconception predictor of HDP across all models (aOR, 3.1; 95% CI, 2.8–3.5, adjusted model) (Table 3). Maternal report of a prepregnancy diagnosis of diabetes (aOR 2.3; 95% CI, 1.7–3.0) was also associated with elevated risk of HDP. Reported prepregnancy advice about improving health was associated with increased risk of HDP (aOR, 1.1; 95% CI, 1.0–1.3). The only maternal behavior associated with decreased risk of HDP was teeth cleaning in the year before pregnancy (aOR, 0.8; 95% CI, 0.7–0.9).

## Discussion

Women’s health status in the year before conception (women’s prepregnancy BMI and prepregnancy diabetes for HDP) were associated with elevated risk of GDM and HDP. However, maternal behaviors in the year before pregnancy did not predict GDM or HDP. This finding suggests that improving perinatal health may require interventions more than a year before conception, with an emphasis on cardiometabolic and mental health. For example, evidence exists that bariatric surgery reduces risk of gestational diabetes; however, women are recommended to wait at least 12 months before conceiving following surgery (24). Thus, bariatric surgery is not a viable intervention for women seeking to conceive within a year, but it may be for women seeking to conceive in 2 or more years. Alternatively, the set of commonly measured preconception behaviors may be insufficient to capture interventions that would improve preconception health and subsequent maternal outcomes. Receiving prepregnancy advice about improving health was associated with increased risk, suggesting that primary care and OB/GYN providers accurately target high-risk women for advice. However, although providers may accurately identify women for advice in the year before pregnancy, it may be unrealistic for women to make sufficient changes in the year before conception to reduce risk. Given the socioeconomic and racial and ethnic disparities in GDM and HDP diagnoses, structural and social interventions more than 1 year before pregnancy may be necessary for prevention.

As previous authors have reported, racial/ethnic and socioeconomic disparities exist in experiences of GDM or HDP and in preconception health indicators (25). Disparities in preconception indicators were minor for health behaviors and greatest for indicators of health care access (eg, insurance, check-up) and prepregnancy health status (eg, obesity, diabetes, hypertension), and non-White women were less likely to have optimal preconception health. Our results regarding the prevalence of GDM and HDP are consistent with other estimates based on older data (26).

Our study has limitations. First, PRAMS collects data on pregnancy outcomes and prepregnancy health retrospectively. We attempted to correct for this by using information from the birth certificate on GDM and HDP, which is more accurate (22). However, our study design precludes assessment of causality. Second, in addition to measuring behaviors retrospectively, information about prepregnancy behaviors is vague. For example, drinking once in the 3 months before pregnancy and drinking daily may represent different levels of risk, which we are unable to capture. Future studies should consider prospective measurement of behaviors of interest. Third, PRAMS is only able to capture whether women report a diagnosis of depression before pregnancy. Thus, women who experienced depression but did not seek care were not included. Prospective measurement of depressive symptoms would better characterize the relationship between preconception depression and HDP. Finally, PRAMS captures whether women had a prepregnancy diagnosis of hypertension, diabetes, or depression but not whether the disease was controlled (by medication, diet, or otherwise) or not. This information could be critical in understanding whether screening before pregnancy has the potential to reduce risk for women with chronic disease. Evidence for diabetes suggests that control of diabetes before pregnancy reduces risk of adverse outcomes, including preeclampsia (16). However, whether control of hypertension would prevent GDM is unknown.

Our study also has strengths. First, PRAMS was conducted on a representative sample of women who gave birth to live infants in participating states for 2016–2017, representing 83% of live births in the United States for included years (21). Second, use of PRAMS allows for comparison of preconception health indicators across a range of domains with existing recommendations for state monitoring of preconception health (3).

These results support previous calls to improve all women’s health across the life course in addition to the focus on the year before conception (1,5,27). Although some preconception health indicators identified women at higher risk, none were promising candidates for intervention in the year before pregnancy. For example, prepregnancy obesity was strongly associated with elevated risk of both HDP and GDM. However, interventions to promote weight loss through diet or exercise among women planning to conceive in the next year have been unsuccessful at preventing GDM or HDP (14,15), which suggests that intervening to prevent or reverse obesity more than a year before women intend to conceive may be necessary to reduce risk of HDP and GDM. Additionally, in the PRAMS sample, only 60% of women planned their pregnancy. Thus, broader structural interventions to improve women’s health may have a greater impact than targeted interventions for women who plan to conceive in the next year. Measuring these indicators in the year before pregnancy describes the status of preconception health in a population; however, to improve preconception health, we must act longer before pregnancy. Many policy models exist to improve overall health outcomes in all women of reproductive age (eg, Medicaid expansion under the Affordable Care Act, Federally Qualified Health Centers) (27). Structural interventions are known to decrease the number of maternal deaths and may similarly improve maternal outcomes such as GDM or HDP, although evidence is limited. For example, states that expanded Medicaid under the Affordable Care Act experienced declines in maternal mortality rates following expansion (28). Conversely, states with fewer Planned Parenthood clinics or more restrictive abortion policies have higher maternal mortality rates (29). Policies to improve access may also decrease racial and ethnic disparities in pregnancy outcomes, as one study of infant mortality showed (30). Our study did not identify behavior changes in the year before pregnancy associated with decreased risk of HDP or GDM. This finding suggests that, although focusing on the year before conception may be a useful motivator for some women to improve their health, expanding, effectively implementing, and evaluating interventions to improve access to and utilization of care across the life course will be necessary to improve maternal outcomes.
